# Three-dimensionally printed surface features to anchor endoluminal spring for distraction enterogenesis

**DOI:** 10.1371/journal.pone.0200529

**Published:** 2018-07-12

**Authors:** Nhan Huynh, Genia Dubrovsky, Joshua D. Rouch, Andrew Scott, Elvin Chiang, Tommy Nguyen, Benjamin M. Wu, Shant Shekherdimian, Thomas M. Krummel, James C. Y. Dunn

**Affiliations:** 1 Department of Surgery, Division of Pediatric Surgery, David Geffen School of Medicine, University of California, Los Angeles, Los Angeles, California, United States of America; 2 Department of Surgery, Division of Pediatric Surgery, School of Medicine, Stanford University, Stanford, California, United States of America; 3 School of Engineering, Department of Bioengineering, University of California, Los Angeles, Los Angeles, California, United States of America; 4 Department of Bioengineering, School of Medicine, Stanford University, Stanford, California, United States of America; Michigan State University, UNITED STATES

## Abstract

Spring-mediated distraction enterogenesis has been studied as a novel treatment for short bowel syndrome (SBS). Previous approaches are limited by multiple surgeries to restore intestinal continuity. Purely endoluminal devices require a period of intestinal attachment for enterogenesis. The purpose of this study is to modify the device to prevent premature spring migration in a porcine model. Two models were created in juvenile mini-Yucatan pigs for the placement of three-dimensionally printed springs. (1) Two Roux-en-y jejunojenostomies with two Roux limbs were made. A spring with bidirectional hooked surface features was placed in one Roux limb and a spring with smooth surface was placed in the other Roux limb. (2) The in-continuity model had both hooked and smooth surface springs placed directly in intestinal continuity. Spring location was evaluated by weekly radiographs, and the intestine was retrieved after 2 to 4 weeks. Springs with smooth surfaces migrated between 1 to 3 weeks after placement in both porcine models. Springs with bidirectional hooked surface features were anchored to the intestine for up to 4 weeks without migration. Histologically, the jejunal architecture showed significantly increased crypt depth and muscularis thickness compared to normal jejunum. Bidirectional features printed on springs prevented the premature migration of endoluminal springs. These novel spring anchors allowed for their endoluminal placement without any sutures. This approach may lead to the endoscopic placement of the device for patients with SBS.

## Introduction

Short bowel syndrome (SBS) is a disease of the small intestine caused by inadequate intestinal length, which leads to poor nutritional absorption and total parental nutrition dependence complicated by infections and liver failure [1]. Healthcare costs associated with this condition exceed $3 billion USD annually [2–3]. SBS is a result of extensive intestinal resection from necrotizing enterocolitis, intestinal atresia, and midgut volvulus [4]. Current surgical procedures to increase intestinal surface area and slow intestinal transit have had limited success [4–7]. New therapeutic modalities are needed.

Distraction enterogenesis to lengthen existing intestines has been studied to address the underlying issue of SBS. Multiple devices including hydraulic pistons, saline injections, and anchored screws have been successful in the application of distractive forces to lengthen intestines in animal models [8–14]. Our laboratory demonstrated effective intestinal lengthening using spring-mediated distractive devices [15–21]. However, these models are limited by multiple surgeries to repeat intestinal lengthening, restore intestinal continuity, or require extraluminal attachments [15,18,20,22–23].

Minimally invasive or endoscopically placed spring devices can avoid such limitations and facilitate clinical application for SBS. Purely endoluminal devices require a period of intestinal attachment for enterogenesis [15]. Spring designs must overcome the digestive tract’s intrinsic properties to contract and expel foreign material into the feces. Similar issues of esophageal and colonic stent migration are noted in other areas of the digestive tract [24–26]. Previously, we developed a high-friction surface spring that evenly distributes distractive forces along the intestinal wall to delay spring detachment, but premature spring migration remained a challenge [15].

Three-dimensional (3D) printing has been widely utilized to create medical models and devices [27–29]. Resolution of 3D-printers is as small as 16 microns [30]. Sophisticated 3D-printing technology can print surface features directly on the springs and optimize surface structures to enhance spring attachment. The purpose of this study is to modify the surface of springs to adequately anchor springs to intestines in a porcine model.

## Materials and methods

### Ethics statement

The use of animals was approved by the University of California, Los Angeles Animal Research Committee (Institutional Review Board Number 2014-142-03 and 2016-002-02A). Vaporized isoflurane was used for anesthesia and phenobarbital was used for euthanasia. All materials were FDA approved for use in humans.

### Spring production and characteristics

Spring models were designed on Fusion Autodesk 360 (Autodesk, San Rafael, CA). Spring model files were sent for 3D-printing to a commercialized manufacturer (Sculpteo, Villejuif, France). A selective laser sintering (SLS) process was used to print the springs [31].The 3D-printed spring was made from nylon, a biocompatible polymer used in sutures [32].

**Fig 1:**
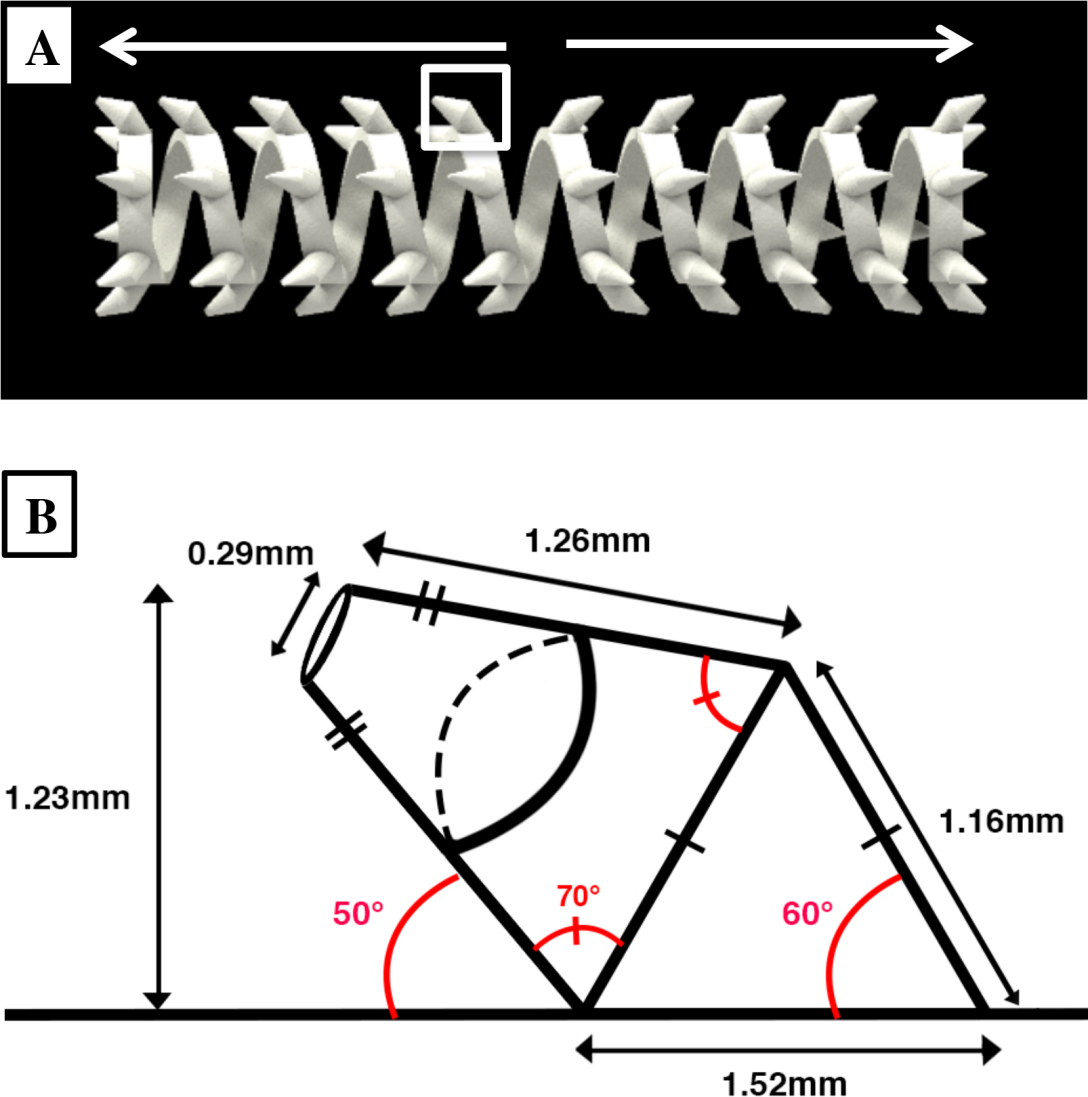
Image of three-dimensionally printed spring with angled bidirectional surface features. (A) Direction of features is indicated by the arrows. One of the surface hooks is isolated by a box. Scale bar is 5 mm. (B) Zoomed in side view schematic of isolated surface feature and corresponding dimensions.

Spring characteristics were extrapolated from previous study of spring with high-friction villi-like surface features in pigs [15]. 3D-printed spring surface features were designed with bidirectional angulated structures to hook onto the intestinal wall (Fig 1). Spring dimensions measured 13 to 14 mm in outer diameter, compressed length of 25 mm, and expanded length of 50 to 70 mm. Smooth surface springs were used for comparison. The springs were placed into size 13 Gelatin capsules (Torpac, Inc, Fairfield, NJ) and were coated three times with cellulose acetate phthalate for delayed expansion (Eastman Chemicals, Kingsport, TN) [33].

### Surgical procedure

Female juvenile mini-Yucatan pigs *Sus scrofa* (S & S Farms), 4 to 6 weeks old, weighing at least 5 kg were intubated, anesthetized with inhaled oxygen, and vaporized isoflurane (n = 9). A midline laparotomy incision was used to enter the abdomen. Two animal models were created.

#### Roux-en-y jejunojenostomies

**Fig 2:**
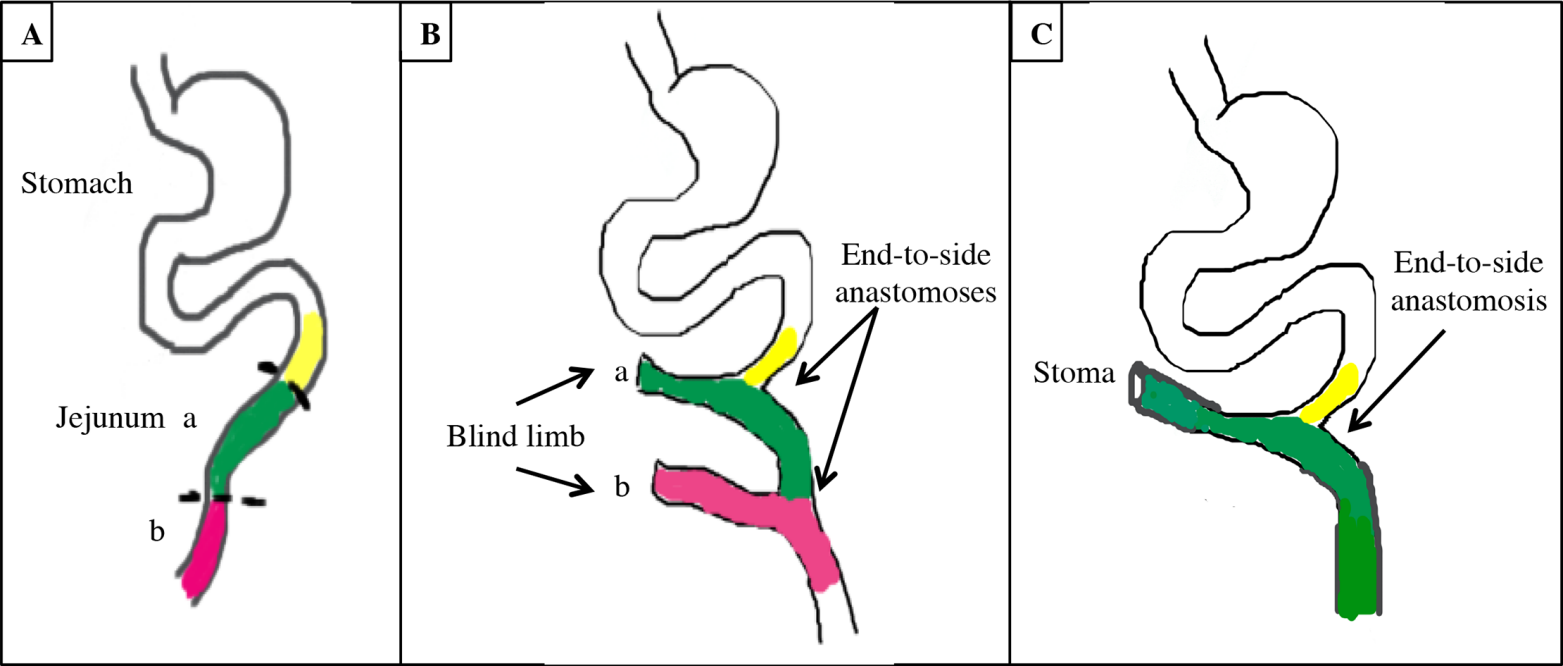
Schematic of Roux-en-y model. (A) Jejunum prior to surgery: *a* is proximal jejunum, *b* is distal jejunum; (B) Roux-en-y jejunojejunostomies with two blind jejunal limbs and end-to-side anatomoses; (C) stoma model with Roux limb and end-to-side anastomosis.

**Fig 3:**
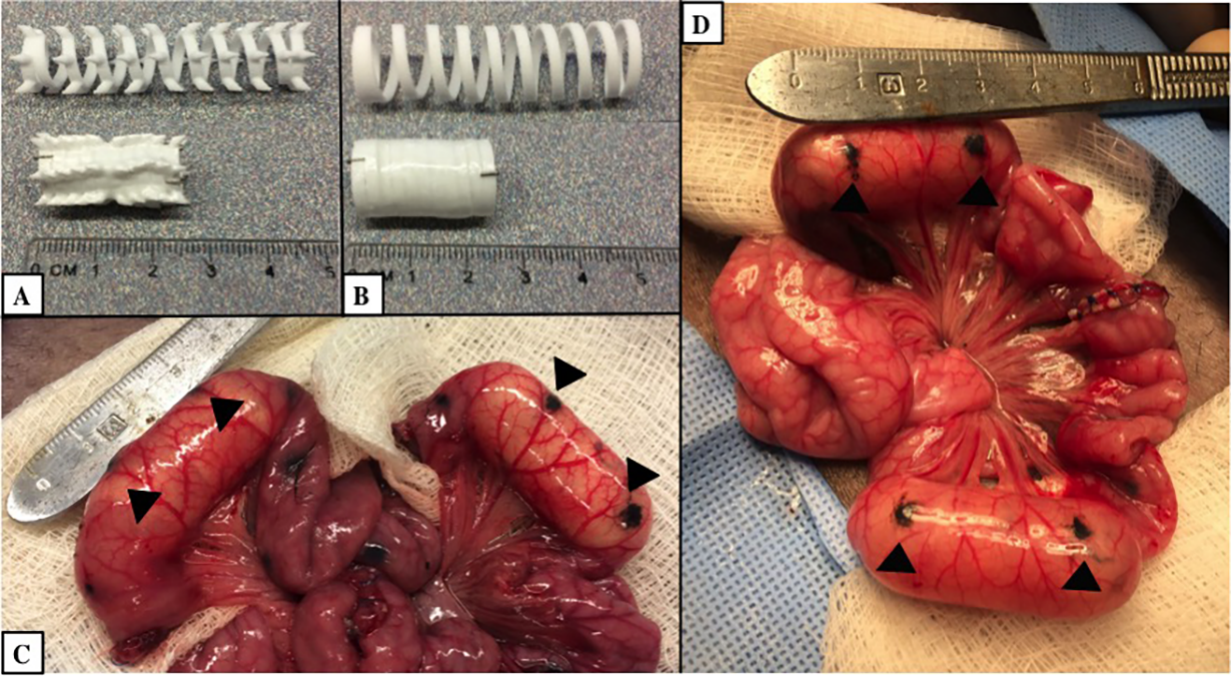
Image of springs in Roux-en-y and in-continuity models. (A) Representative photos of expanded and compressed springs with hooked surface features and (B) smooth surface. (C) Operative image showing Roux-en-y configuration with two blind Roux limbs with the hooked spring placed in the Roux limb on the left of the image and smooth spring placed in the Roux limb on the right of the image. (D) Operative image showing in-continuity model with hooked spring proximal to anastomosis and smooth spring distal to anastomosis. Arrowheads mark areas of India ink injected at the ends of the compressed springs.

Two Roux-en-y jejunojenostomies with two 30-cm defunctionalized blind end Roux limbs were created in the proximal jejunum. The jejunum was transected 30 cm from the ligament of Trietz to create the first Roux limb. The second Roux limb was created 30 cm from the first jejunojenostomy (n = 4) (Figs 2 and 3). In two animals, one Roux limb was made into a stoma for sequential spring placement (Fig 2). An encapsulated 3D-printed spring with surface features was inserted in one limb and an encapsulated smooth surface spring was inserted in the other limb (Fig 3). The Roux limb ends were closed with 4–0 Prolene suture (Ethicon, Johnson & Johnson; Somerville, NJ) in simple interrupted fashion. The jejunojenostomies were repaired with end-to-side anastomoses with 4–0 Prolene in simple interrupted fashion to restore intestinal continuity.

#### In-continuity spring placement

A subset of animals (n = 3) underwent in-continuity spring placement. The jejunum was transected 30 cm from the ligament of Trietz. An encapsulated 3D-printed spring with hooked surface features was placed proximal to the transected jejunum. An encapsulated 3D-printed smooth surface spring was inserted distal to the transected jejunum (Fig 3). The transected jejunum was repaired with an end-to-end anastomosis with 4–0 Prolene suture in a simple interrupted fashion to restore intestinal continuity.

**Fig 4:**
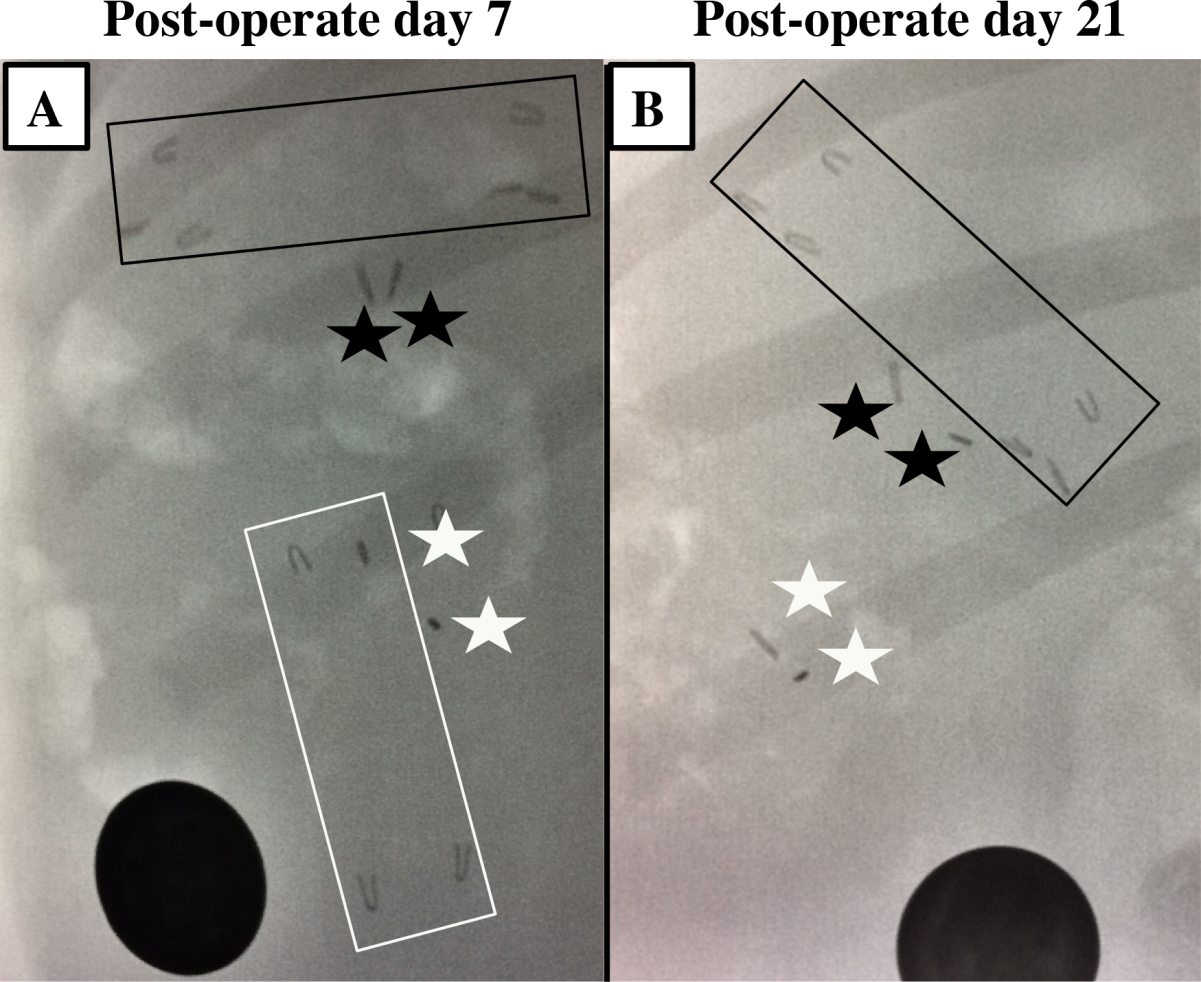
Abdominal radiographs of spring placement. Radiographs show metal clips at the ends of the spring and on adjacent mesentery on (A) post-operative day 7 and (B) post-operative day 21. Hooked spring is labeled with three metal clips at each end, shown in black box. Smooth surface spring is labeled with two metal clips at each end, shown in white box. The smooth surface spring had migrated by post-operative day 21. Stars mark the mesenteric clips. Scale object measurement is 2.1 cm in diameter.

In both animal models, metal clips were placed on each end of the spring and on the mesentery adjacent to the spring ends in order to monitor spring expansion on weekly abdominal x-rays (Fig 4). India ink (Becton, Dickinson and Company, Franklin Lakes, NJ) was injected at the ends of the compressed spring to evaluate initial location of springs upon specimen retrieval. The bowel was placed back into the abdomen and the abdominal wall was closed in layers.

Animals with in-continuity springs were placed on a fortified liquid diet. Animal weights were recorded. Animals were euthanized with phenobarbital and intestinal segments were retrieved after 2, 3, and 4 weeks for final assessment of spring location.

### Histologic analysis

Jejunum marked with India ink and normal jejunum were fixed in 10% buffered formalin overnight followed by embedding in paraffin. Normal jejunum used for comparison was jejunum in the Roux limb without the hooked spring in the Roux-en-y model and jejunum without the hooked spring in the in-continuity model. Tissue was aligned in perpendicular cross sections. Tissue blocks were cut into 5 μm sections and stained with hematoxylin and eosin. Sections were examined and recorded at 4x and 10x magnification using light microscopy (Olympus Corporation, Waltham, MA). Muscularis propria thickness and crypt depth were measured for each specimen.

### Statistical analysis

Data were expressed as mean values ± standard deviations. Two-tailed, paired and unpaired Student’s t-tests were used for statistical analyses where appropriate.

## Results

Animals tolerated the procedure well without signs of obstruction or perforation. Roux-en-y animals demonstrated weight gain of 137 ± 42 grams per day. In-continuity animals gained 83 ± 20 grams per day.

### Duration of spring attachment

**Fig 5:**
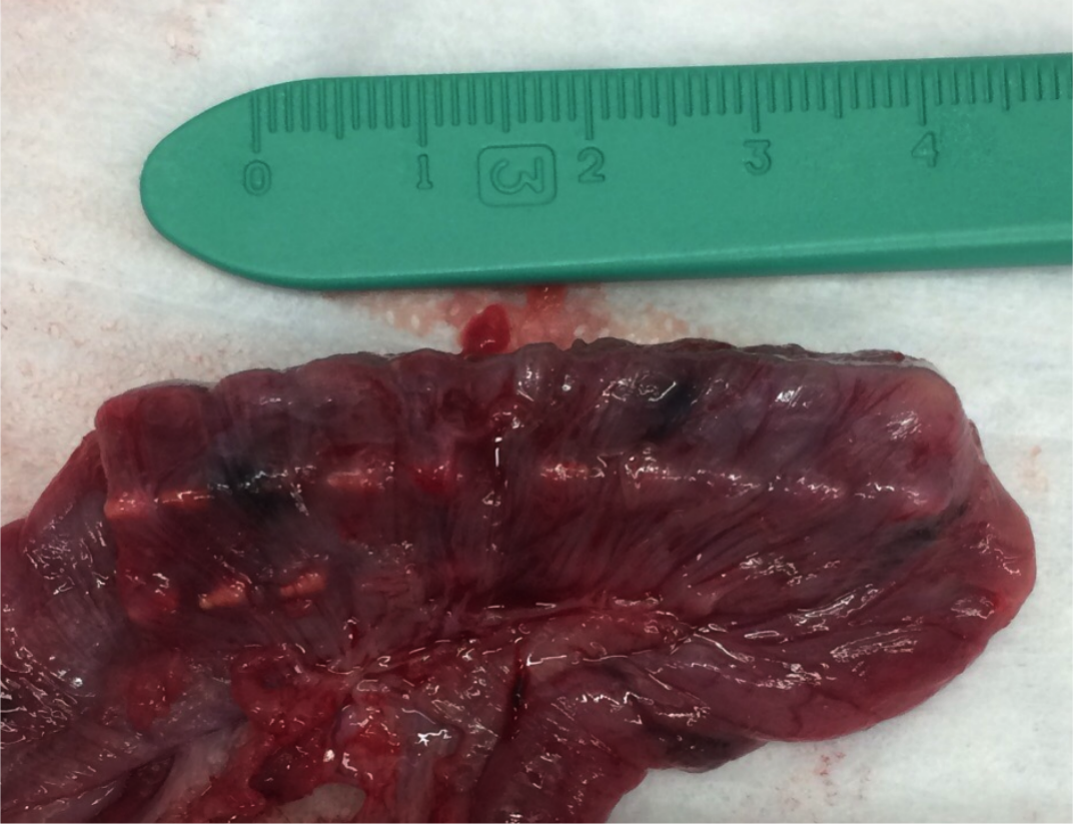
Intestinal segment at time of specimen retrieval. Intestinal segment with fully expanded hooked spring at time of specimen retrieval on post-operative day 28.

All springs with smooth surfaces migrated from the initial location into the fecal stream between 1 to 3 weeks after placement. Springs with bidirectional surface features were anchored to the intestine for the entire duration without migration as evaluated by weekly abdominal radiographs. These findings were confirmed at time of specimen retrieval at 2, 3 and 4 weeks after placement (Fig 5).

### Histological analysis

**Fig 6:**
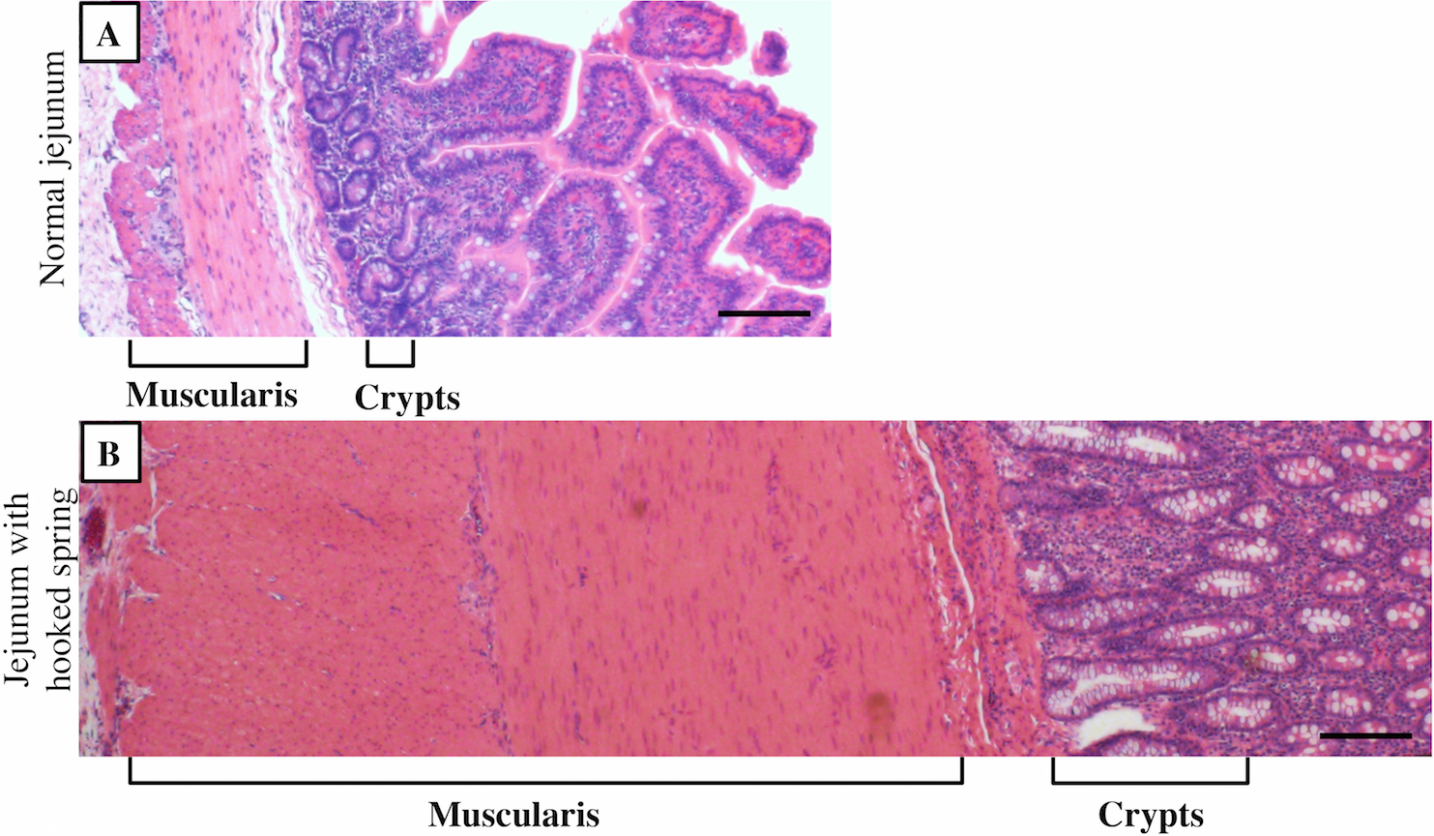
Histology of jejunum with spring versus normal jejunum. Jejunal segments with hooked spring demonstrate significantly increased crypt depth and muscularis propria thickness relative to normal jejunum without spring. Light microscopy images of hematoxylin and eosin-stained (A) normal jejunum without spring and (B) jejunum with hooked spring in-continuity at 4× magnification. Scale bar is 100 μm.

Histologically, jejunal architecture was preserved in intestinal segments with hooked 3D-printed springs. Roux limb jejunum with hooked springs showed significantly increased crypt depth (438±106 μm versus 204±43μm; p = 0.001) and muscularis thickness (782±332μm versus 230±128μm; p = 0.01) in comparison to Roux limb jejunum without hooked springs. In-continuity jejunum with hooked springs showed a greater degree of increased crypt depth in comparison to jejunum without hooked springs (656±46μm versus 353±86μm; p = 0.006). Muscularis thickness of these segments was similarly thickened (733±164μm versus 362±60μm; p = 0.02) (Fig 6).

## Discussion

Previous anchoring techniques employed a high-friction surface on the spring to evenly distribute distractive forces along the intestinal length to delay spring detachment [15]. In this study, spring surface features were modified to further delay premature spring migration. This novel spring with enhanced spring-to-bowel coupling is suitable for endoscopic delivery and negates the need for complex anchoring techniques or additional surgeries for sequential spring placement.

Spring-mediated distraction enterogenesis successfully demonstrated intestinal lengthening in animal models with a few barriers to clinical application. The animal models and spring characteristics have evolved to address these limitations. Prior to this study, an isolated jejunal segment model was utilized to study spring-mediated intestinal lengthening [17–18]. Although lengthening was 3-fold in rodents, the clinical impact was limited by tissue loss during restoration into continuity and multiple surgeries [18]. Roux-en-y jejunojenostomy was then used as a model for repeated spring placements but required extraluminal attachments to anchor springs [20]. The in-continuity model was developed to avoid extensive bowel manipulation and multiple surgeries [15]. Although this model was safe and feasible, premature spring migration prevented adequate time for maximal distraction enterogenesis [15]. Addition of a high-friction surface delayed spring migration however full-thickness sutures were still required to anchor the spring [15]. This current study successfully demonstrated that angulated, bidirectional surface features enhanced spring attachment and prevented spring migration.

Bidirectional surface features were designed to accommodate the dynamic back and forth motion of peristalsis. Hooks in each direction assist in anchoring the spring in place. The hooked springs are delivered intraluminally inside a smooth gelatin capsule to avoid damage to intestinal epithelium. Once the capsule dissolves, the hooks on the spring engage to the intestinal wall.

The Roux-en-y jejunojejunostomy model was used to study the effectiveness in anchoring the hooked spring. The two limbs allowed for an internal control spring to be studied simultaneously with the hooked spring. We recognize that other surgical models are more telling of potential clinical application [15,22]. Once the hooked features demonstrated adequate anchoring, the novel spring was placed via a defunctionalized stoma and allowed for sequential manual spring insertion via stoma without additional surgeries needed for spring placement. The spring did not migrate. To avoid excessive bowel manipulation and evaluate the potential for endoscopic spring placement, springs were inserted in direct enteric continuity and demonstrated no spring migration. Further studies in these prototypical animal models is needed to conclude effectiveness but these preliminary studies is proof-of-concept that the novel hooked spring can be delivered in a minimally invasive fashion, endoscopically, or manually via stoma in a repeatable fashion.

Although these nylon springs are biocompatible, it is not biodegradable. Previous studies used polycaprolactone (PCL) to create biodegradable springs to avoid a procedure to retrieve the spring at a later time [18]. 3D-printers with PCL printing capabilities do not have sufficient resolution to produce the small surface features at this time, and non-degradable materials were used for this study as a proof-of-concept. Previous studies have shown that springs eventually detached and spontaneously passed in the fecal stream without causing complications; thus a biodegradable material may not be necessary. The hooked spring has spring constant of 45 N/m, which is three times that of the PCL spring used in our previous studies. The larger spring constant may lead to faster expansion of the spring once the gelatin capsule dissolves before the necessary engagement of the spring to the bowel could take place. Nevertheless, the hooks allowed the spring to remain in place albeit in an expanded state. The spring constant is determined by the material and physical aspects of the spring including the width of the band of the spring. Currently the width is larger than previous PCL springs to accommodate the base of the hooks. Additional spring designs are under investigation to adjust the spring constant.

The current spring does not show significant intestinal lengthening. While desirable, intestinal lengthening is not the focus of this study. Optimizing spring characteristics to enhance distraction enterogenesis is under ongoing investigation.

Normal jejunum was used as the comparison to analyze histological changes. Therefore, histological changes can be attributed to the effect of the spring on the intestines. Histology of retrieved jejunum demonstrates increased muscularis thickness and crypt depth, which are characteristic features of tissue subjected to distractive force [11,13]. Increased muscularis thickness and crypt depth are well-documented in the literature in labs who study distraction enterogenesis. Nearly all models evaluating use of distraction enterogenesis have reported these histological changes [8–11, 13, 15–22, 34–35]. Previous studies demonstrated cellular proliferation in lengthened segments in association with these histological changes, intact enzymatic function, absorption capability, and intact barrier function [11, 18]. The role of mechanotransduction pathways has been proposed as the mechanism responsible for distraction enterogenesis. Mechanical force is transmitted via cell membrane integrins that activates the FAK pathway leading to downstream signaling that results in cellular proliferation and decreased apoptosis [36, 37]. Additionally, increase in insulin-like growth factor (IGF-I) expression has been localized to the muscularis propria of lengthened segments and could account for thickened muscularis [38]. In prior studies, intestinal motility and absorptive function remained intact despite muscularis thickening in these animals [19]. Other studies have also demonstrated return to normal muscularis thickness once the physical force is removed [39].

Crypt depth in in-continuity jejunum is significantly increased in comparison to the crypt depth in defunctionalized Roux limbs. Greater degree of crypt depth increase in in-continuity jejunum is likely from exposure to alimentary nutrition. These observations suggest that the mechanical force imparted by the springs result in cellular proliferation, even in the absence of intestinal lengthening. This approach may yield new therapeutic options via the introduction of mechanical loading in the setting of short bowel syndrome.

## Conclusion

In summary, bidirectional hooked spring surface features optimized spring to bowel wall coupling and prevented spring migration. Such novel spring anchors permit purely endoluminal spring placement without any sutures. Repeated endoscopic spring placements or manual insertion via stoma is possible. We believe this approach may lead to the clinical application of the device for patients with short bowel syndrome in the future.

## Supporting information

S1 File Data of springs and histological findings. (XLSX)

## References

[1] Reinshagen K, Adams R, Trunk M, Wessel LM. The chronic liver disease in patients with short bowel syndrome: Etiology and treatment. Minerva Pediatr 2009;61:273–81. 19461571

[2] Wales PW, De Silva N, Kim J, Lecce L, To T, Moore A. Neonatal Short Bowel Syndrome: Population-Based Estimates of Incidence and Mortality Rates. J. Pediatr. Surg., vol. 39, 2004, p. 690–5. 10.1016/j.jpedsurg.2004.01.036 15137001

[3] Spencer AU, Kovacevich D, McKinney-Barnett M, Hair D, Canham J, Maksym C, et al Pediatric short-bowel syndrome: The cost of comprehensive care. Am J Clin Nutr 2008;88:1552–9. 10.3945/ajcn.2008.26007 19064515

[4] Squires RH, Duggan C, Teitelbaum DH, Wales PW, Balint J, Venick R, et al Natural history of pediatric intestinal failure: Initial report from the pediatric intestinal failure consortium. J Pediatr 2012;161 10.1016/j.jpeds.2012.03.062 22578586 PMC3419777

[5] Thompson JS, Weseman R, Rochling F a, Mercer DF. Current management of the short bowel syndrome. Surg Clin North Am 2011;91:493–510. 10.1016/j.suc.2011.02.006 21621693

[6] Bianchi A. Longitudinal intestinal lengthening and tailoring: results. J R Soc Med 1997;90:429–32. 10.1055/s-2008-1072258 9306995 PMC1296456

[7] Kim HB, Fauza D, Garza J, Oh JT, Nurko S, Jaksic T. Serial transverse enteroplasty (STEP): A novel bowel lengthening procedure. J. Pediatr. Surg., vol. 38, 2003, p. 425–9. 10.1053/jpsu.2003.50073 12632361

[8] Park J, Puapong DP, Wu BM, Atkinson JB, Dunn JCY. Enterogenesis by mechanical lengthening: Morphology and function of the lengthened small intestine. J Pediatr Surg 2004;39:1823–7. 10.1016/j.jpedsurg.2004.08.022 15616941

[9] Safford SD, Freemerman a J, Safford KM, Bentley R, Skinner M a. Longitudinal mechanical tension induces growth in the small bowel of juvenile rats. Gut 2005;54:1085–90. 10.1136/gut.2004.061481 15840689 PMC1774906

[10] Puapong DP, Wu BM, Lam MM, Atkinson JB, Dunn JCY. Distension enterogenesis: Increasing the size and function of small intestine. J Pediatr Surg 2006;41:763–7. 10.1016/j.jpedsurg.2006.02.017 16567190

[11] Spencer AU, Sun X, El-Sawaf M, Haxhija EQ, Brei D, Luntz J, et al Enterogenesis in a clinically feasible model of mechanical small-bowel lengthening. Surgery 2006;140:212–20. 10.1016/j.surg.2006.03.005 16904972 PMC1764912

[12] Chen Y, Zhang J, Qu R, Wang J, Xie Y. An animal experiment on short gut lengthening. Chin Med J (Engl) 1997;110:354–7. 9594302

[13] Shekherdimian S, Panduranga MK, Carman GP, Dunn JCY. The feasibility of using an endoluminal device for intestinal lengthening. J Pediatr Surg 2010;45:1575–80. 10.1016/j.jpedsurg.2010.03.015 20713203

[14] Fisher JG, Sparks EA, Khan FA, Dionigi B, Wu H, Brazzo J, et al Extraluminal distraction enterogenesis using shape-memory polymer. J. Pediatr. Surg., vol. 50, 2015, p. 938–42. 10.1016/j.jpedsurg.2015.03.013 25812443

[15] Huynh N, Rouch JD, Scott A, Chiang E, Wu BM, Shekherdimian S, et al Spring-mediated distraction enterogenesis in-continuity. J Pediatr Surg 2016;51 10.1016/j.jpedsurg.2016.09.024 27692863

[16] Scott A, Rouch JD, Huynh N, Chiang E, Shekherdimian S, Lee SL, et al Mechanical lengthening in multiple intestinal segments in-series. J Pediatr Surg 2016;51 10.1016/j.jpedsurg.2016.02.058 27013424

[17] Rouch JD, Huynh N, Scott A, Chiang E, Wu BM, Shekherdimian S, et al Scalability of an endoluminal spring for distraction enterogenesis. J Pediatr Surg 2016;51 10.1016/j.jpedsurg.2016.09.023 27665493

[18] Sullins VF, Wagner JP, Suwarnasarn AT, Lee SL, Wu BM, Dunn JCY. A novel biodegradable device for intestinal lengthening. J Pediatr Surg 2014;49:109–13. 10.1016/j.jpedsurg.2013.09.040 24439592

[19] Sullins VF, Wagner JP, Walthers CM, Chiang EK, Lee SL, Wu BM, et al Function of mechanically lengthened jejunum after restoration into continuity. J Pediatr Surg 2014;49:971–5. 10.1016/j.jpedsurg.2014.01.033 24888845

[20] Sullins VF, Scott A, Wagner JP, Steinberger D, Lee SL, Wu BM, et al Intestinal lengthening in an innovative rodent surgical model. J Pediatr Surg 2014;49:1791–4. 10.1016/j.jpedsurg.2014.09.022 25487485

[21] Rouch JD, Scott A, Jabaji ZB, Chiang E, Wu BM, Lee SL, et al Basic fibroblast growth factor eluting microspheres enhance distraction enterogenesis. J Pediatr Surg 2016;51:960–5. 10.1016/j.jpedsurg.2016.02.065 26995517

[22] Demehri FR, Utter B, Freeman JJ, Fukatsu Y, Luntz J, Brei D, et al Development of an endoluminal intestinal attachment for a clinically applicable distraction enterogenesis device. J. Pediatr. Surg., vol. 51, 2016, p. 101–6. 10.1016/j.jpedsurg.2015.10.026 26552895 PMC4713322

[23] Demehri FR, Wong PM, Freeman JJ, Fukatsu Y, Teitelbaum DH. A novel double-balloon catheter device for fully endoluminal intestinal lengthening. Pediatr Surg Int 2014;30:1223–9. 10.1007/s00383-014-3612-9 25319796

[24] Verschuur EML, Homs MY V, Steyerberg EW, Haringsma J, Wahab PJ, Kuipers EJ, et al A new esophageal stent design (Niti-S stent) for the prevention of migration: A prospective study in 42 patients. Gastrointest Endosc 2006;63:134–40. 10.1016/j.gie.2005.07.051 16377330

[25] Sims K, Payne I, Grimm L Jr. Esophageal stent migration requiring colonoscopic retrieval. Surg Endosc Other Interv Tech 2016;30:S342–S342.

[26] Han S, Lee JH. Colonic Stent-Related Complications and Their Management. Clin Endosc 2014;47:415–9. 10.5946/ce.2014.47.5.415 25325000 PMC4198557

[27] Zein NN, Hanouneh IA, Bishop PD, Samaan M, Eghtesad B, Quintini C, et al Three-dimensional print of a liver for preoperative planning in living donor liver transplantation. Liver Transplant 2013;19:1304–10. 10.1002/lt.23729 23959637

[28] Kurenov SN, Ionita C, Sammons D, Demmy TL. Three-dimensional printing to facilitate anatomic study, device development, simulation, and planning in thoracic surgery. J Thorac Cardiovasc Surg 2015;149:973–979.e1. 10.1016/j.jtcvs.2014.12.059 25659851

[29] Hollander J, Genina N, Jukarainen H, Khajeheian M, Rosling A, Makila E, et al Three-Dimensional Printed PCL-Based Implantable Prototypes of Medical Devices for Controlled Drug Delivery. J Pharm Sci 2016;105:2665–76. 10.1016/j.xphs.2015.12.012 26906174

[30] "Objet Connex 3D Printers", 2010. Objet Printer Solutions. Retrieved 2012-01-31.

[31] Chung H, Das S. Functionally graded Nylon-11/silica nanocomposites produced by selective laser sintering. Mater Sci Eng A 2008;487:251–7. 10.1016/j.msea.2007.10.082

[32] Israelsson LA, Jonsson T. Closure of midline laparotomy incisions with polydioxanone and nylon: The importance of suture technique. Br J Surg 1994;81:1606–8. 10.1002/bjs.1800811114 7827883

[33] Stark R, Panduranga M, Carman G, Dunn JCY. Development of an endoluminal intestinal lengthening capsule. J Pediatr Surg 2012;47:136–41. 10.1016/j.jpedsurg.2011.10.031 22244406

[34] Shekherdimian S, Scott A, Chan A, Dunn JCY. Intestinal lengthening in rats after massive small intestinal resection. Surgery 2009 8; 146(2): 291–5. 10.1016/j.surg.2009.03.034 19628087

[35] Stark R, Aupekan T, Bondad S, Dunn JCY. Restoration of mechanically lengthened jejunum into intestinal continuity in rats. J Pediatr Surg. 2011 12;46(12):2321–6. 10.1016/j.jpedsurg.2011.09.019 22152874

[36] Sueyoshi R, Ignatoski K, Okawada M, Teitelbaum D. Distraction-Induced Intestinal Growth: The Role of Mechanotransduction Mechanism in a Mouse Model of Short Bowel Syndrome. Tissue Engineering 2014; 20 (3 and 4): 830–841. 10.1089/ten.tea.2013.0383 24070252 PMC3926159

[37] Safford SD, Freemerman AJ, Safford KM, Bentley R, Skinner MA. Longitudinal mechanical tension induces growth in the small bowel of juvenile rats. Gut. 2005 8; 54(8): 1085–1090. 10.1136/gut.2004.061481 15840689 PMC1774906

[38] Dunn JC, Lam MM, Lee E. Increased expression of insulin-like growth factor in intestinal lengthening by mechanical force in rats. J Pediatr Surg. 2007;42(12):2057–2061. 10.1016/j.jpedsurg.2007.08.027 18082707

[39] Scott A, Sullins VF, Steinberger D, Rouch JD, Dunn JCY, et al Repeated mechanical lengthening of intestinal segments in a novel model. J Pediatr Surg. 2015 6;50(6):954–7. 10.1016/j.jpedsurg.2015.03.020 25818320

